# An Incidental Discovery of Amyand’s Hernia: A Case Study and Literature Review on Its Intraoperative Management

**DOI:** 10.7759/cureus.11858

**Published:** 2020-12-02

**Authors:** Pramath Kakodkar, Wee Xuan Neo, Muhammed Hassan Tahir Khan, MN Baig, Tahir Khan

**Affiliations:** 1 School of Medicine, National University of Ireland Galway, Galway, IRL; 2 School of Medicine, National University of Galway Ireland, Galway, IRL; 3 Orthopaedics, University Hospital Galway, Galway, IRL; 4 Department of Vascular Surgery, Mater Private Hospital, Cork, IRL

**Keywords:** amyand’s hernia, amyand, general surgery, hernioplasty, appendectomy variants

## Abstract

Amyand's hernia (AH) is a rare form of an inguinal hernia where the vermiform appendix is found within the hernia sac. Diagnosis is usually based on incidental finding intraoperatively. The AH makes up a small proportion of all inguinal hernia cases, and concurrent acute ischemic complication makes up an even smaller subset.

We present an 85-year-old male who was referred to general surgery services for a growing mass on his right lower quadrant in the inguinal region. This was non-tender on palpation, and therefore there was no suspicion of ischaemic complications. An open hernioplasty was performed with resection of the appendix. The AH in this patient would be conventionally classified as type 1 AH, which would be managed with hernial reduction and mesh repair. The anatomical variance in our patient's AH increased the risk for hernial incarceration; hence an appendectomy was also performed despite the absence of acute appendicitis. This approach was also deemed necessary to avoid the recurrence of hernia due to its large size and adhesions within the hernial sac.

This study reports a novel management approach for an incidentally discovered type 1 AH. It highlights that there is a lack of management guidance for the AH anatomical variants. The classification and management for AH under the conventional Losanoff and Basson's AH classification model have limitations that can be amended by incorporating the physical dimensions of the AH. This approach will enable surgeons to recognize and manage more variations of AH while mitigating downstream complications.

## Introduction

Amyand’s hernia (AH) is an intriguing surgical finding of the appendix in the inguinal sac. The incidence of AH spans from 0.4% to 1% of all generic hernias operated upon by the general surgery services [1]. Most patients with AH often present asymptomatically, making its diagnosis arduous. Occasionally, AH patients can present with downstream complications such as acute appendicitis due to incarceration, strangulation, phlegmon formation, or perforation within the inguinal sac. These acute ischemic complications tend to be rare and present at a frequency of 0.1% [2]. The majority of AH is discovered incidentally during hernia repair operations. Early diagnostic imaging usage can warrant a rapid preoperative diagnosis of AH [3]. Furthermore, the preliminary clinical presentation of AH comprises tender inguinal swelling, which diagnosticians can perceive as bowel strangulation or volvulus. Hence, AH carries a large burden of misdiagnosis [4].

Due to the heterogeneity in the presentation of Amyand’s hernia, there exist three definitions: 1) a groin hernia with an incidental finding of the appendix in the hernia sac; 2) a groin hernia with a strangulated appendix requiring specific concerns when dealing with an incarcerated hernia; 3) primary appendicitis protruding into the groin hernia defect. This recognition of the appropriate definition of AH for each individual case presentation will dictate the optimal management strategy. This case report highlights how a patient with a large-sized AH was managed with an open hernioplasty and appendectomy in the absence of appendicitis. Furthermore, the existing literature on the classification and management of AH was also reviewed to provide insights on the limitations in the management of AH anatomical variants.

## Case presentation

We present the case of an 85-year-old male who was referred to our general surgery department for further investigations due to an irreducible inguinal hernia. The patient noted the sensation of a gradually increasing mass on his right lower quadrant. These findings were not accompanied by changes in bowel habits, but there was an increase in his nocturnal urinary frequency to twice daily. Abdominal and genito-urinal examination revealed a soft, non-tender mass extending into the inguinal-scrotal region. The Valsalva maneuver while standing upright revealed an evident protrusion of the hernia in the right inguinal region. Examination of the contralateral inguinal-scrotal region revealed an absence of pathology. Due to the non-tender course of the hernia, no diagnostic imaging was ordered. A diagnosis of an indirect inguinal hernia was made. Furthermore, given that the hernia was irreducible, there was a concern of impending hernial incarceration.

Management

**Figure 1:**
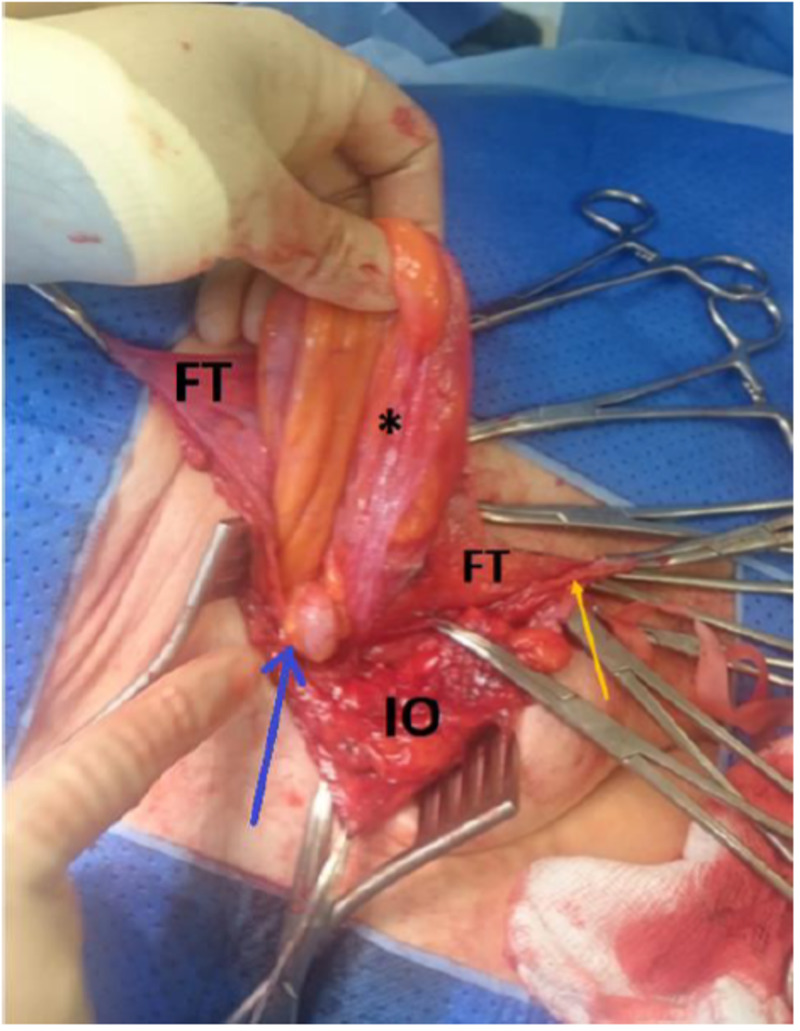
Hernia sac with an incarcerated appendix (*) outpouching from the deep inguinal ring (blue arrow), which is visible on reflection of Internal oblique (IO) and fascia transversalis (FT). The ilioinguinal nerve (yellow arrow) is secured.

An elective open mesh repair of the right inguinal hernia was recommended. The open hernioplasty was the chosen management approach instead of a laparoscopic approach due to the dimensions of this anatomically large hernia (6cm x 12cm). Surgical management with a right inguinal repair was commenced with a standard approach of exposing the external oblique until the superficial ring of the inguinal canal. On transecting the aponeurosis of the external oblique, the hernial sac was located (Figure 1).

**Figure 2:**
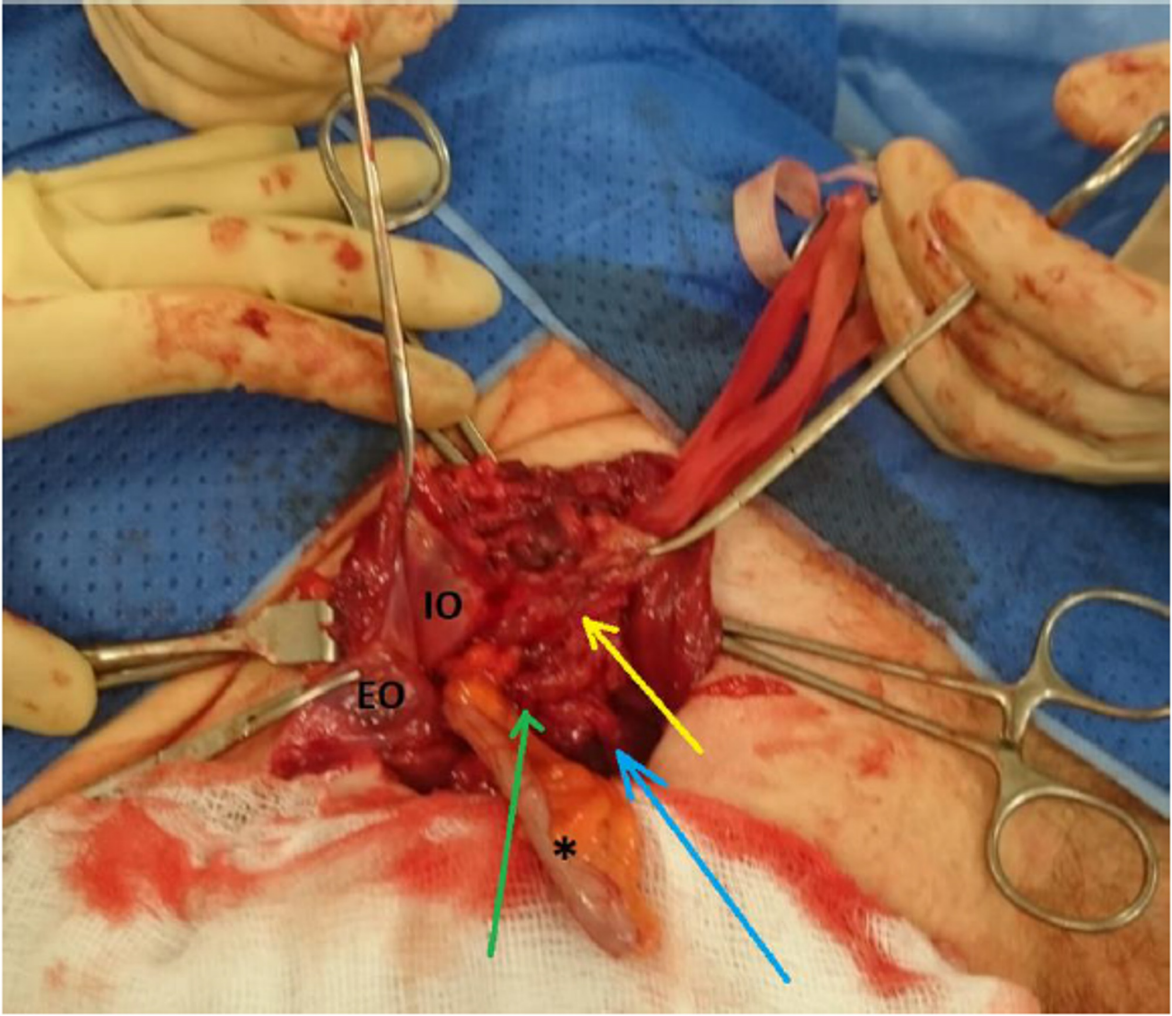
Reflection of the external oblique (EO) and internal oblique (IO) provides inguinal canal exposure. The hernial sac (yellow arrow), the inferior epigastric vessels (green arrow) and the spermatic cord (blue arrow) are seen exit into the superficial inguinal ring. The appendix (*) is seen visibly herniating out of the deep inguinal ring.

**Figure 3:**
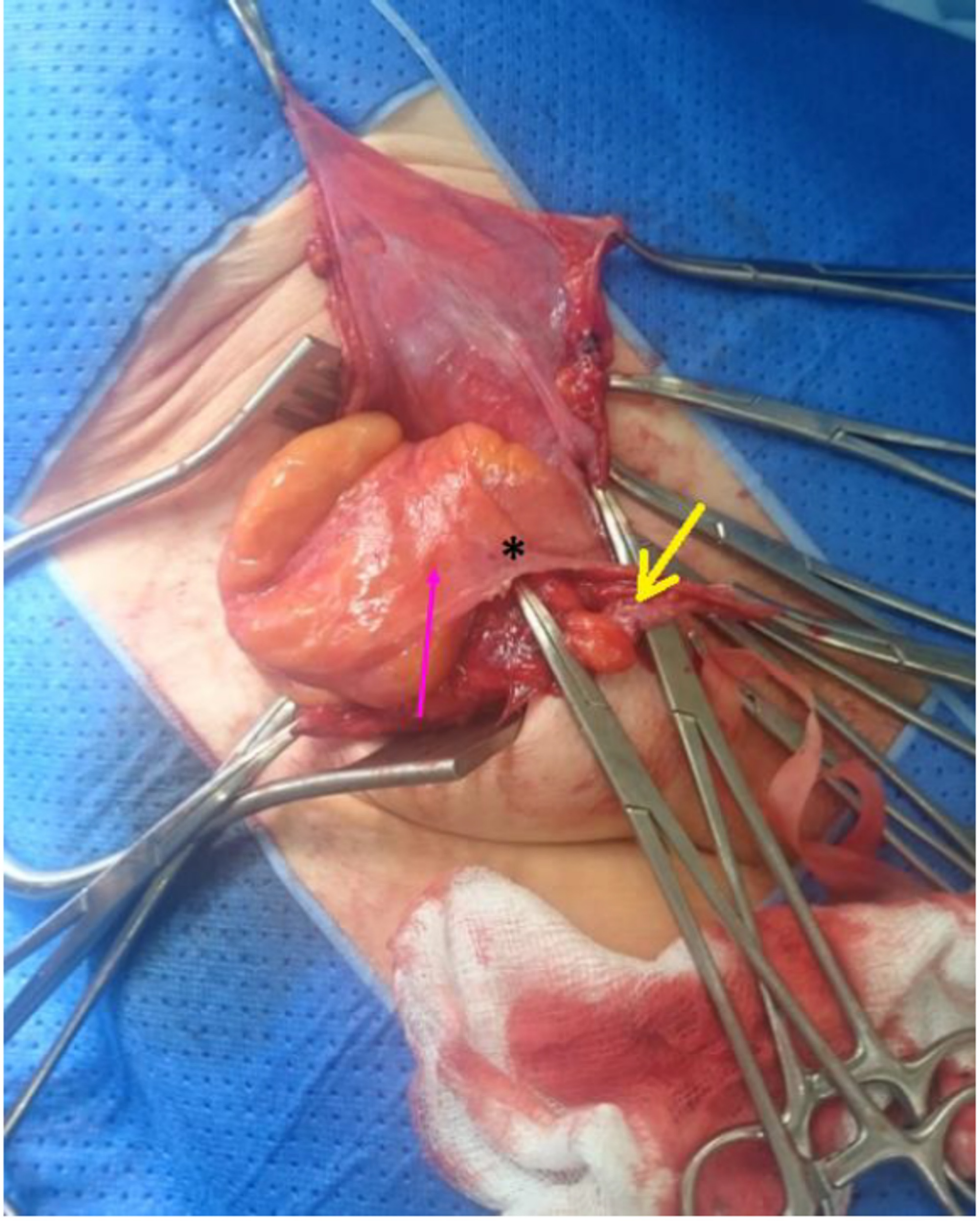
Amyand’s hernia wherein the appendix (*) is enclosed within the hernial sac (pink arrow) and physically adheres to it with the fibroelastic tissue (yellow arrow).

Intraoperatively, the ilioinguinal nerve was identified and secured, after which the cord was separated from the hernial sac (Figures 2-3). Both these figures provide a clearer view of the surgical field and highlight the regional anatomy within the hernial sac. 

**Figure 4:**
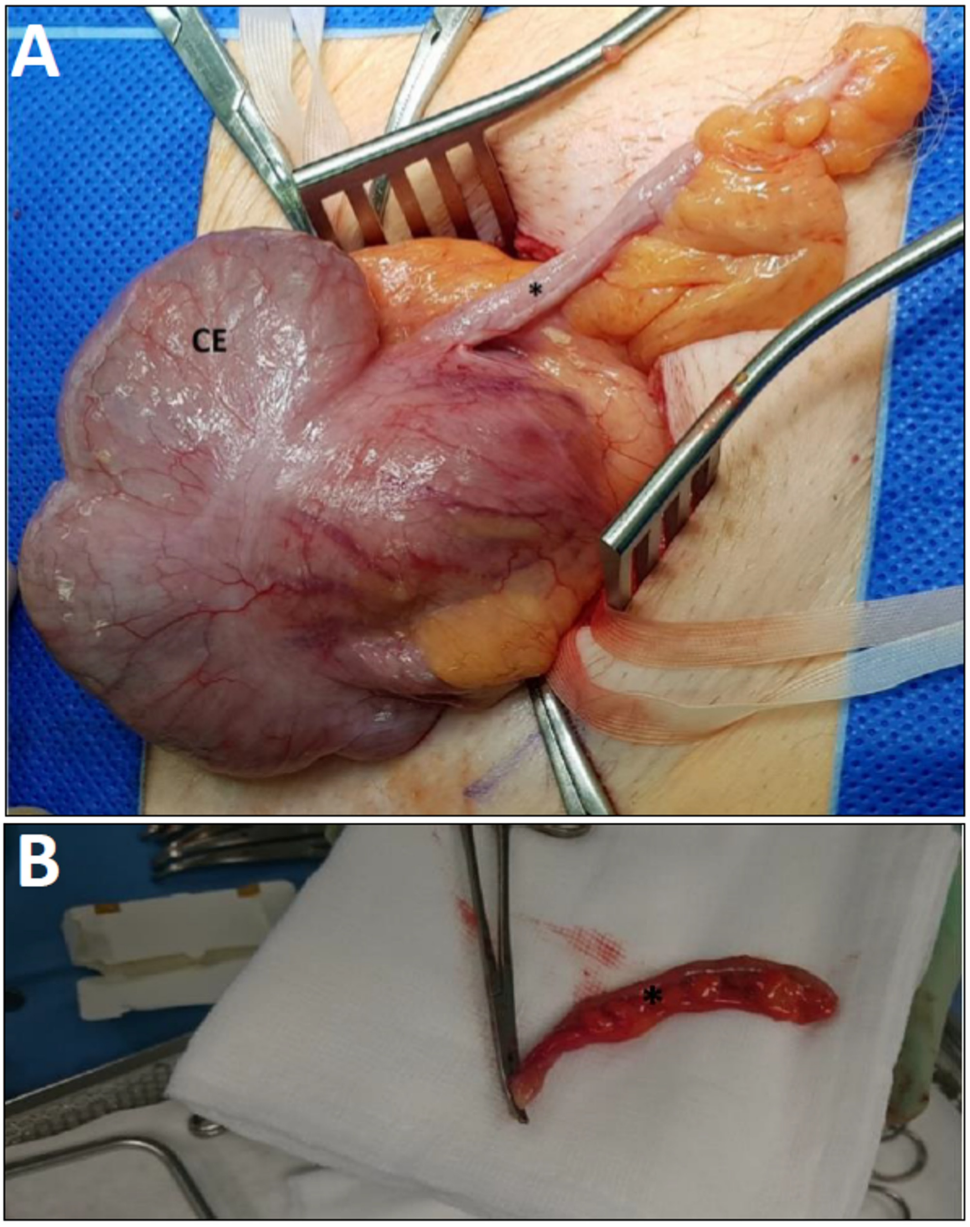
A: shows the opened hernial sac contents revealing the caecum (CE) and the appendix (*). B: shows the resected gross specimen of the appendix (*) with no visible localized inflammatory changes.

The contents of the hernial sac revealed the incidental finding of an Amyand’s hernia, i.e., a continuous cord of the caecal appendix spanning across the hernial sac (Figure 4-A). Furthermore, there were no visible, localized inflammatory changes in the gross resected appendix specimen (Figure 4-B).

The vesicular contents within the opened hernial sac were reduced back into the peritoneal cavity. This reduction was transfixed with a 2-O Vicryl® suture and was consequently repaired with an Ultrapro® mesh. Both 2-O Prolene® and Vicryl sutures were used to shelve across a region of 6 cm by 12 cm. A standard closure without any complications was completed.

Outcome and follow-up

The patient was discharged after 24 hours due to the absence of postoperative complications and had an uneventful follow-up at two weeks. The Clavien-Dindo classification was scored as 0 on the postoperative day 30.

## Discussion

The field of appendicectomy was first pioneered by the French-born British general surgeon Claudius Amyand in the 1700s. Furthermore, in 1735, Amyand successfully operated on an inflamed appendix encased within the hernial sac of an 11-year-old male child in St. George's Hospital London [5]. This was the first case of the successful surgical management of Amyand’s hernia with appendectomy and herniotomy that was documented in the Royal Society of London’s philosophical transactions.

The current guidelines for the management of an AH is an algorithm that is heavily dependent on the extent of the pathology of the appendix incarcerated within the hernia sac. Table 1 summarizes the findings of the Losanoff and Basson classification system that describes the recommended surgical management options for the different types of Amyand’s hernia [6]. The fundamental approach gleans on the surveillance of the integrity of the appendix within the inguinal hernia and the corresponding downstream complications of uncontrolled acute appendicitis such as peritonitis and abdominal sepsis.

**Table 1:** Adapted from Losanoff and Basson’s Classification and Management of AH

Classification type	Appendix status	Systemic status	Surgical management
1	Normal	-	Hernia reduction + mesh repair
2	Acute appendicitis	No sepsis	Appendectomy + primary non-mesh repair
3	Acute appendicitis	Peritoneal sepsis	Laparotomy, appendectomy + primary non-mesh repair
4	Acute appendicitis	Abdominal pathology	Manage as type 1-3. Background abdominal pathology must be explored.

There is a general agreeability for the management type 3 and 4 AH, but this system fails to account for the hernia size. The dimensions of the hernia impose practical operative restrictions on the maneuvering range and the axis for laparoscopy. Furthermore, suture repair in large hernia poses a massive degree of tension upon the omental closure line [7]. Therefore, hernioplasty with mesh repair was deemed to be an appropriate management option for this case.

In 2017, Kose et al. discovered an anatomical variant of AH that contests Losanoff and Basson’s management guidelines for type 1 and 2 [8]. This urges for creating a new classification system that is inclusive of management guidelines for all Amyand’s hernia types. Our case was like that of Kose et al., the appendiceal cord was surrounded by a fibroelastic band that physically anchored it to the hernia sac (Figure 3). Therefore, an appendectomy had to be performed to enable structural reduction of the hernia despite the absence of visible signs of acute appendicitis. Conventional classification of this Amyand’s hernia would be type 1, yet the corresponding management recommendations from Losanoff and Basson’s are devoid of the inclusion of an appendectomy to reduce the hernia [9]. This rationale behind not performing an appendectomy in AH patients is based on studies that performed autopsies and revealed that the AH can remain asymptomatic throughout the life course, and hence does not require resection [10, 11].

Our recommendation for performing an appendectomy in the absence of acute appendicitis was also proposed by Quartey et al. in the first reported case of an incarcerated recurrent AH. In this study, the AH was first classified as Losanoff type 1; hence appendectomy was not performed, then it precipitated into an incarcerated AH [12]. This was more recently described by Kose et al. as well. This publication investigated the management of five similar Amyand’s hernia patients and deemed that appendectomies adjunct to the mesh hernioplasty was the preferred method of management [8]. All these patients proceeded to recovery without any postoperative complications and had no incidence of recurrence of a hernia.

The decision to perform an appendectomy in this case despite the absence of acute appendicitis was heavily dependent on the senile age of the patient (84 years), and the adherence of the appendix to the hernia sac via the fibroelastic band. Literature has shown that emergency laparotomy or laparoscopy has morbidity and mortality as high as 21% in patients older than 65 years [13]. The expected primary outcome of this management approach was to avoid the future risk of recurrence of herniation and mitigate the risk of postoperative infection. The historical norm for indications to perform an appendectomy in AH was constrained due to the iatrogenic risk of secondary appendicitis and an increased propensity for peritoneal adhesions. In Losanoff and Basson's model, this schema of management is preferable, as there is a lower risk of infection compared to the risk of hernia recurrence with a primary repair only.

Another limitation in the Losanoff and Basson's model is the management of type 2 Amyand's hernia with an appendectomy adjunct with a non-mesh repair. Multiple studies have questioned the validity of the non-mesh repair approach in type 2 Amyand's hernia management, as there is a documented increased incidence of postoperative complications associated with it [14-16]. The absence of coverage in the classification of the anatomical variants of Amyand's hernia and the added postoperative complications with non-mesh repair makes the current standardized guidelines obsolete. Chatzimavroudis et al. discussed the usage of propylene plug placement in a case of incarcerated recurrent inguinal hernia with acute appendicitis, stating that while the decision for a mesh hernia repair may appear hazardous prima facie, this can be addressed by postoperative administration of 3-5 days to prevent mesh infection and thus, the concern of a septic environment (i.e. strangulated or incarcerated inguinal hernia) should not be considered as an absolute contraindication for prosthetic implantation [17]. This is corroborated by Torino et al. suggesting that adequate antibiotic irrigation of the inguinal region with an aponeurotic drain could allow for feasible and safe usage of a synthetic mesh [18]. Hence, it is important to consider amending Losanoff and Basson’s classification of Amyand’s hernia.

In diagnosis, Lombardo et al. reported that diagnosis is almost always made intraoperatively, regardless of any clinical findings of appendicitis, and that ultrasound of groin and scrotum would not yield any additional preoperative information [19]. However, we believe that suspicion of Amyand's hernia can be confirmed preoperatively using an ultrasound (US) or an abdominal CT (CT-Abdo). Expected findings on the US include the presence of an elongated tubular cord that is blind-ended, containing a thick adventitial lining, and is communicating with the caecum while being encapsulated within the hernia sac. On a CT-Abdo, the expected finding pertains to the discovery of a tubular cord that is blind-ended with a proximal origin from the caecum, and the distal portion is invaginating into an inguinal hernia sac [1]. In asymptomatic patients, AH is an incidental find and therefore is a need to include diagnostic imaging criteria in classifying asymptomatic irreducible inguinal hernia to rule out AH preoperatively. An ideal solution would have been a standardized guideline for classification and surgical management of AH that incorporates a scoring system to evaluates variables such as the age of the patient, clinical examination, and diagnostic imaging status of the appendix.

However, the lack of consensus on the true definition of AH remains an impediment to the formation of such guidelines. The heterogeneous clinical presentation of AH would prime the surgeon for different operative procedures of either an appendicectomy or hernia repair, given the propensity for asymptomatic AH to be discovered as an incidental finding without any prior imaging. With laparoscopic approaches becoming increasingly frequent in modern practice for complicated abdominal surgery such as local peritonitis and strangulated hernia, surgeons would have to be not only prepared to perform both appendicectomy and hernia repair in the same operation without prior planning but also exercise clinical judgment in navigating the individual variance in operative anatomy.

## Conclusions

In this case report, the definition of Amyand’s hernia (AH) that fit was a type of inguinal hernia wherein the appendix is incarcerated within the hernial sac. This presentation is yet another case of an incidental intraoperative finding of the AH. There is still no methodical standardization for preoperative diagnosis in asymptomatic patients due to the epidemiological scarcity. Considering ultrasound imaging in a large-sized inguinal hernia even if it is reducible may be beneficial in mitigating the downstream complications. The current guidelines lack inclusion for the classification and management of atypical presentations of AH. There is an increased need for prospective investigatory studies in this area to facilitate the diagnostic predictability for general surgeons to rule out AH on suspicion of any asymptomatic or symptomatic inguinal hernias.
